# Cartilage Tissue Engineering by Extrusion Bioprinting: Process Analysis, Risk Evaluation, and Mitigation Strategies

**DOI:** 10.3390/ma14133528

**Published:** 2021-06-24

**Authors:** Mauro Petretta, Giovanna Desando, Brunella Grigolo, Livia Roseti

**Affiliations:** 1 RegenHU Ltd., Z.I. du Vivier 22, 1690 Villaz-ST-Pierre, Switzerland; mauro.petretta@regenhu.com; 2 Rizzoli RIT-Research, Innovation & Technology Department, IRCCS Istituto Ortopedico Rizzoli, SSD RAMSES, Via di Barbiano 1/10, 40136 Bologna, Italy; livia.roseti@ior.it

**Keywords:** FMEA/FMECA, bioprinting, cartilage regeneration, translational research

## Abstract

Extrusion bioprinting is considered promising in cartilage tissue engineering since it allows the fabrication of complex, customized, and living constructs potentially suitable for clinical applications. However, clinical translation is often complicated by the variability and unknown/unsolved issues related to this technology. The aim of this study was to perform a risk analysis on a research process, consisting in the bioprinting of a stem cell-laden collagen bioink to fabricate constructs with cartilage-like properties. The method utilized was the Failure Mode and Effect Analysis/Failure Mode and Effect Criticality Analysis (FMEA/FMECA) which foresees a mapping of the process to proactively identify related risks and the mitigation actions. This proactive risk analysis allowed the identification of forty-seven possible failure modes, deriving from seventy-one potential causes. Twenty-four failure modes displayed a high-risk level according to the selected evaluation criteria and threshold (RPN > 100). The results highlighted that the main process risks are a relatively low fidelity of the fabricated structures, unsuitable parameters/material properties, the death of encapsulated cells due to the shear stress generated along the nozzle by mechanical extrusion, and possible biological contamination phenomena. The main mitigation actions involved personnel training and the implementation of dedicated procedures, system calibration, printing conditions check, and, most importantly, a thorough knowledge of selected biomaterial and cell properties that could be built either through the provided data/scientific literature or their preliminary assessment through dedicated experimental optimization phase. To conclude, highlighting issues in the early research phase and putting in place all the required actions to mitigate risks will make easier to develop a standardized process to be quickly translated to clinical use.

## 1. Introduction

Bioprinting of three-dimensional (3D) cartilage-like structures composed of scaffolds, cells, and soluble factors has a huge potential for clinical application [1,2]. The promising results from preclinical studies provided new perspectives for orthopedics since cartilage repair still represents a major unsolved clinical need [3]. Articular hyaline cartilage damage or loss can arise at any age due to trauma, rheumatic disease, or just wear and tear [4]. This tissue displays a low self-repair ability, and untreated lesions tend to get worse over time leading to whole joint degeneration and consequently prosthetic replacement. None of the traditional treatments can restore cartilage structure and function satisfactorily [5,6,7]. In recent times, regeneration approaches foresaw the development of engineered tissues, composed of scaffolds (e.g., collagen and hyaluronic acid), cells (chondrocytes or mesenchymal stromal cells (MSCs)), and soluble factors (e.g., Transforming Growth Factor -β and Insulin Growth Factor -1) to mimic the heterogeneous composition of cartilage and its biomechanical properties [8,9]. By mimicking typical extracellular matrix (ECM) features, scaffolds with improved bioactive and biodegradable properties represent suitable microenvironments where MSCs can exert their secretory activities through the release of anti-inflammatory, immunomodulatory, and regenerative mediators [10]. Their crosstalk with the cells within the lesion site and the surrounding tissues can ensure controlled processes of cartilage tissue formation and remodeling [11].

Bioprinting represents a novel approach in the biomedical field since it allows the simultaneous printing of cells and scaffold by creating a 3D tissue-like structure additively, in a layer-by-layer manner, from a predefined 3D computer model obtained from the patient’s medical images [12,13]. This procedure has the great advantage of ensuring a more punctual control of the engineered structure in terms of architecture, complexity, cell distribution, and chemical composition [1]. Altogether, these features guarantee a precise match between implant and defect size, thereby enabling to develop customizable structures depending on the patient’s clinical need [14].

Despite encouraging results in the 3D bioprinting technology, there is still a gap between the research applications and their clinical use due to several issues that need to be addressed [15]. Optimizing and standardizing this process in the research phase is pivotal for fostering its proper and faster translation into clinics [16]. To the best of our knowledge, no studies have described, in a step-by-step fashion, the sequential phases of a bioprinting process with its respective failure modes and mitigation actions. This study aims to narrow this gap by performing an innovative, proactive risk analysis of a cartilage bioprinting process through an interdisciplinary approach. Highlighting biological issues in an early phase will provide new perspectives for implementing the most standardized processes and speeding up their future clinical translation.

## 2. Materials and Methods

The method utilized was the qualitative/quantitative Failure Mode and Effect Analysis/Failure Mode and Effect Criticality Analysis (FMEA/FMECA). The FMEA/FMECA technique is a proactive risk analysis, based on the analysis of a process, aimed at identifying and preventing possible errors of the system before they occur. It is composed of five steps: choice of the critical process; establishment of a working team; mapping of the selected process; risk analysis (qualitative and quantitative); and development and implementation of improvement actions and monitoring of outcomes [17].

### 2.1. Choice of the Process

To choose the critical process to be analyzed, several factors were considered.

(i) Process diffusion in terms of literature data, agency reports, lab presence, the potential for translation into clinical practice, or already known clinical applications.
(ii) Process complexity in terms of time length, number of steps, involved operators, and complex technologies.
(iii) Severity of possible harmful events/reagents.
(iv) Recurrent issues in the lab related to the process.

### 2.2. Team Composition

A multidisciplinary team was made up to enclose operators with knowledge and experience of the process but presenting different expertise and roles. Evaluations were also based on literature data and current legislation. The team met on several occasions and worked with established deadlines.

### 2.3. Process Mapping and Product Quality Evaluation

#### 2.3.1. Process Mapping

A clear and detailed description of all process stages was provided. In particular, the team divided the process into phases, each phase in steps, and each step into simpler activities.

#### 2.3.2. Product Quality Evaluation

Criteria for product quality evaluation were based on the desired outcome of the bioprinting process. Failure to meet any criteria would imply that the product does not have the desired quality level, and the need to determine how to adjust and optimize the process. Since product quality represents the end goal, we did not consider its assessment procedures as part of the risk analysis, but a setpoint to consider for identifying the different failure modes and their consequences.

### 2.4. Risk Analysis

#### 2.4.1. FMEA/FMECA

For each activity of the process, potential failure modes (human errors, instrumentation/device defects, or issues related to materials/reagents that might compromise the process and/or the product) were identified. Potential causes and consequences (effects) were also described [14].

The identified failure modes were characterized by three variables: the likelihood of occurrence (O), the severity of the potential effects (S), and the chance of detection before affecting process/product quality (D). Variables were quantified using specific 1–10 ranking scales (Table 1, Table 2 and Table 3) obtained by adjusting previously employed ones [1,18] to the 3D bioprinting context. By multiplying O × S × D, the Risk Priority Number (RPN) was calculated.

Using the chosen criteria, the highest possible RPN value is 1000 (10 × 10 × 10) and the lowest is 1 (1 × 1 × 1). There is no best practice or rule for setting an RPN threshold limit; however, this could be useful to prioritize the mitigation actions to apply. We set an RPN threshold limit of 100, which is a frequently utilized value in healthcare environments [19].

#### 2.4.2. Improvement Actions and Monitoring

The improvement action plan was constructed according to the RPN values. The team, therefore, decided the timing for the monitoring and the improvement actions adopted.

## 3. Results

### 3.1. Choice of the Process

Pneumatic extrusion-based bioprinting of an MSC-laden collagen 3D scaffold was chosen for the FMEA-FMECA analysis considering the aspects reported below:

(i) Extrusion bioprinting—which uses viscous gels/pastes—does not need a melting step, thus allowing cell surviving and embedding. For this reason, most commercial bioprinters are based on this technology [20]. Moreover, it has proved to be far more reliable for tissue engineering applications because of its ability to mimic the structural and functional complexity of cartilage tissue. We selected a collagen type I-based hydrogel as bioink, which has been demonstrated to be suitable for cartilage tissue engineering applications by improving cell viability, attachment, proliferation, and homing [21]. Moreover, due to the low immunogenicity, it has been already utilized in clinical investigations [22,23]. Regarding cell component, MSCs were selected thanks to their unique properties: (a) the ability to self-renew and to differentiate toward several phenotypes including the chondrogenic one; (b) the immune-privileged status; and (c) the ability to release a plethora of active factors capable of modulating the local microenvironment (paracrine activity).
(ii) 3D bioprinting is a novel, multistep, and complex technology. The main drawbacks are a relatively low resolution and deformation [24,25,26].
(iii) Due to the presence of embedded live cells, generally, bioprinting processes do not involve harmful/toxic reagents. Using thermosensitive materials such as collagen could enable to get rid of chemical or UV activated crosslinking processes affecting cell survival [27]. We considered these risks in the FMEA/FMECA because these processes may be adopted in some applications, as reported in the discussion paragraph. It is also to be considered that, in certain cases, collagen bioink is stored in a solution of acetic acid and neutralized immediately before their combination with cells [27].
(iv) The death of encapsulated cells due to the high level of shear stresses generated along the nozzle may represent a recurrent issue. This risk is strongly reduced by (a) the use of low viscosity natural polymers, such as collagen, that can undergo quick gelation or crosslinking process post-deposition; and (b) by the adoption of ad hoc solutions for the process (e.g., increased needle diameter or use of conical needles) [13].

### 3.2. Team Composition and Timetable

The team was composed of the authors of this publication: the lab risk manager; a bioengineer specialized in 3D bioprinting technology; a biologist expert in MSC culture and tissue engineering; and the person in charge of the lab with broad expertise in personalized regenerative medicine and clinical translation. The team meetings covered approximately three months.

### 3.3. Process Mapping and Product Quality Evaluation

#### 3.3.1. Process Mapping

**Figure 1:**
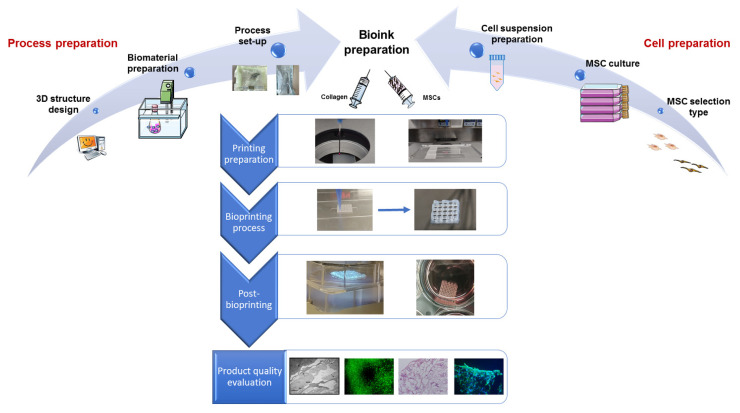
Schematic sequential representation of process phases.

The process object of the FMEA/FMECA is described in Figure 1. Briefly, it begins with the creation of a digital model through a Computer-Aided Design (CAD) approach. Specific algorithms convert the digital model into a series of instructions for construct creation via Computer-Aided Manufacturing (CAM). Lately, the machine extrudes through a nozzle (extrusion-print-head) an MSC-laden collagen bioink (layer by layer from the bottom to the top), physically creating the construct. In the end, the construct is post-processed to activate eventual crosslinking mechanisms and incubated to guarantee cell survival.

The selected extrusion bioprinting process was divided into three sequential macro-phases: pre-bioprinting, bioprinting, and post-bioprinting.

The pre-bioprinting phase includes two independent sub-phases running “in parallel” until the bioink is prepared: process preparation and cell preparation.

Process preparation encloses three steps:

(i) 3D structure design (4 activities);
(ii) Biomaterial preparation (6 activities);
(iii) Process set-up (3 activities).

Cell preparation encloses three steps:

(i) MSC selection type (2 activities);
(ii) MSC culture (3 activities);
(iii) Cell suspension preparation (3 activities).

The bioprinting phase is composed of three steps:

(i) Bioink preparation (4 activities);
(ii) Printing preparation (3 activities);
(iii) Bioprinting process (4 activities).

Post-bioprinting encloses two activities:

(i) Cross-linking;
(ii) Incubation at 37 °C, 95% relative humidity, 5% CO_2_.

#### 3.3.2. Product Quality Evaluation

Key criteria to evaluate product quality are described below:

(i) Printing fidelity, defined as a construct with a well-defined shape and architecture, by matching the fabricated object morphological features to the designed ones. The design should be adapted to provide the constructs with the required transport properties to guarantee nutrient supply, mechanical properties to mimic the native tissue, as well as favoring cell well-being and cartilage ECM production;
(ii) Cell-laden construct shape retention (microscopic, pore, and filament size) and stability over time, both dependent on the selected material properties and the implemented crosslinking mechanisms. Failure in meeting this condition will inevitably result in sample loss;
(iii) Cell viability, affected by shear stresses, environment conditions, and crosslinking mechanisms, which may cause phenotypical and functional changes of cells or death;
(iv) Absence of microbiological contamination causing cell death and microbial growth;
(v) Interaction between cells and scaffold evaluated through specific morphological assessment including scanning electron microscopy (SEM) and histology;
(vi) Cell ability to differentiate toward the cartilage phenotype, dependent on the clinical need, choice of the cell source, cell types, and culture conditions, as well as morphology of the fabricated constructs as specified in point (i).

### 3.4. Risk Analysis

Table 4, Table 5, Table 6 and Table 7 summarize the results of the FMEA/FMECA performed on the different phases of the investigated bioprinting process. Table 4 refers to the pre-bioprinting phase and process preparation sub-phase; Table 5 to the cell preparation sub-phase; Table 6 to the bioprinting phase; and Table 7 to the post-bioprinting phase. In each table, from the left column to the right one, the following are described: steps of the different phases, activities of each step, failure modes identified for each activity, potential causes, possible consequences, assigned values of Occurrence, Severity, and Detection, calculated Risk Priority Number, and mitigation actions.

The team identified a total of 47 failure modes during the whole process: 31 in the pre-bioprinting phase (15 during process preparation sub-phase (Table 4) and 16 during cell preparation sub-phase (Table 5)), 12 in the bioprinting phase (Table 6), and 4 in the post-bioprinting phase. The number of failure modes was slightly higher than that of activities because, for each operation, several issues can be identified. The highest number of failure modes was observed for the pre-bioprinting phase, which is also composed of the highest number of activities.

Potential causes were 71: 44 in the pre-bioprinting phase (29 during process preparation sub-phase (Table 4) and 15 during cell preparation sub-phase (Table 5)), 20 in the bioprinting phase (Table 6), and 7 in the post-bioprinting phase (Table 7). As can be observed in Table 4, Table 6 and Table 7, failure modes and potential causes were mostly due to lack of previous process optimization in terms of material properties identification, cell-material interaction, cell survival, and parameters selection for the whole bioprinting process. Lesser causes are represented by human errors. However, Table 5 shows the most potential failure modes due to human errors during the procedure.

The inability of achieving and/or keeping a defined 3D construct shape, stability, and cell viability are among the most common consequences during the bioprinting process. They may be caused by improper material deposition and stacking or post-printing phenomena, such as swelling or inadequate crosslinking. Further recurrent consequences are related to cell damage or death due to mechanical stresses, adverse process conditions, and microbial contaminations. All the reported consequences would naturally imply the need to repeat the experiment, with the deriving time and economic loss (material and cell waste).

**Figure 2:**
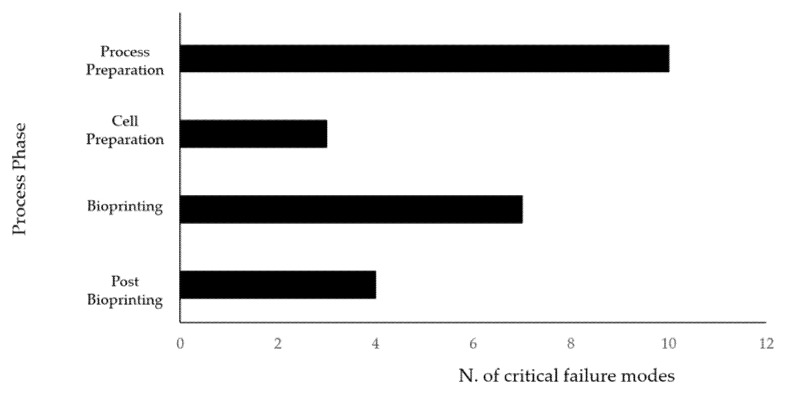
Schematic representation of critical failure modes (RPN > 100) occurrence for the different process phases: process preparation, cell preparation, bioprinting, post-bioprinting.

The calculated RPNs greater than 100 were 24, as reported in Figure 2: 13, in the pre-bioprinting phase (10 during process preparation sub-phase (Table 4) and 3 during cell preparation sub-phase (Table 5)), 7 in the bioprinting phase (Table 6), and 4 in the post-bioprinting phase (Table 7). The higher values of RPN were generally correlated to higher values of severity combined with low detection chances, mostly assigned to failure modes that may cause cell death or microbial contamination.

Mitigation actions involved personnel training and the implementation of dedicated procedures, system calibration, printing conditions check, and, most importantly, a thorough knowledge of biomaterial and cell properties that can be built either through the provided data/scientific literature or their preliminary assessment through a dedicated experimental optimization phase.

**Figure 3:**
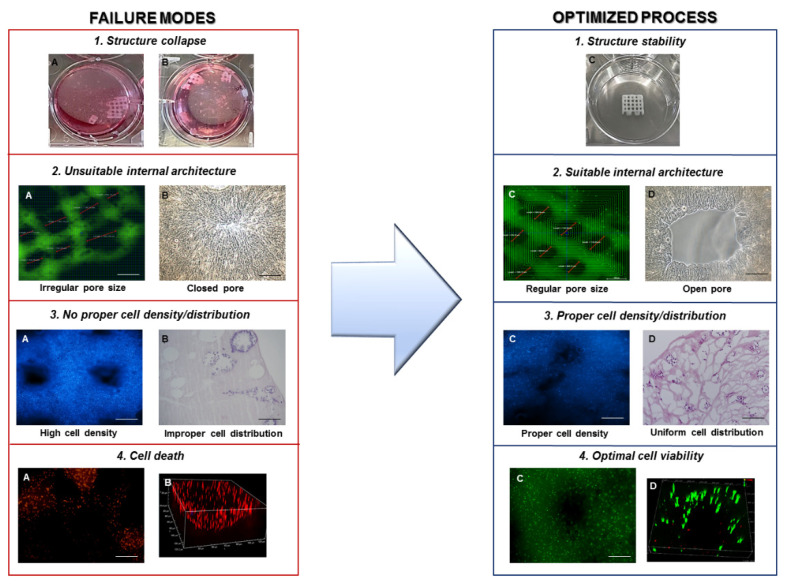
Schematic representation of possible failure modes with their related consequences (left part) and improved outcomes after mitigation actions (right part). (1) Macroscopic images of a scaffold losing shape stability under culture conditions (**A**,**B**; left panel) and scaffold with a stable structure (**C**, right panel). (2) Fluorescence (**A**) and bright-field microscopy (**B**) images of a scaffold presenting irregular/closed pore size (left panel) and suitable pore distribution and shape (**C**,**D**, right panel). (3) Fluorescence microscopy image with DAPI staining (**A**) and histological staining with Hematoxylin/Eosin (**B**) showing an unsuitable (left panel) and suitable cell density and distribution (right panel). (4) Fluorescence microscope image of Live and Dead assay reporting dead cells (red staining) in 2 dimensions (**A**) and after three-dimensional stack (**B**) (left panel) and live cells (green staining) in 2D (**C**) and after 3D stack (**D**) (right panel).

Figure 3 represents some examples of possible failure modes and their related consequences (left part) and the improved outcomes after mitigation actions (right part).

## 4. Discussion

This study applies the FMEA/FMECA to a laboratory experimental procedure. It is a method commonly utilized in healthcare and pharmaceutical systems to assess the risk of failure and identify the most critical areas for improvements [28,29,30]. Although not formally required for non-regulated investigations, FMEA/FMECA offers the great advantage to identify and control errors with the perspective to increase research performance and facilitate clinical translation. This represents an added value when performing the analysis on novel technologies such as 3D bioprinting in orthopedics, also considering the reduced amount of available information. Limitations of the FMEA/FMECA method are represented by the subjectivity of the members of the team; the lack of standardized scales for O, S, and D values; and the use of RPN ones to prioritize mitigation actions. Subjectivity is unavoidable but can be minimized by choosing a heterogeneous team to reduce scale variability. We utilized some O, S, and D values already applied in our institution and planned mitigation actions for each failure mode to monitor the whole process.

We focused our analysis on the extrusion-based bioprinting of an MSC-laden collagen 3D scaffold, a process that is currently under development in our lab for potential future applications in the regeneration of cartilage. We chose a standalone collagen bioink, despite the fact that combination with different materials has been generally considered to improve printability [31,32,33,34]. In parallel, we selected a population of solely human MSCs from bone marrow and not a mixture (i.e., MSCs and chondrocytes), which would better foster regenerative processes in the articular microenvironment [35]. Because of the complexity of the bioprinting technology alongside the critical issues in the standardization, we decided to start analyzing a simple process and only later move to more complex ones.

**Figure 4:**
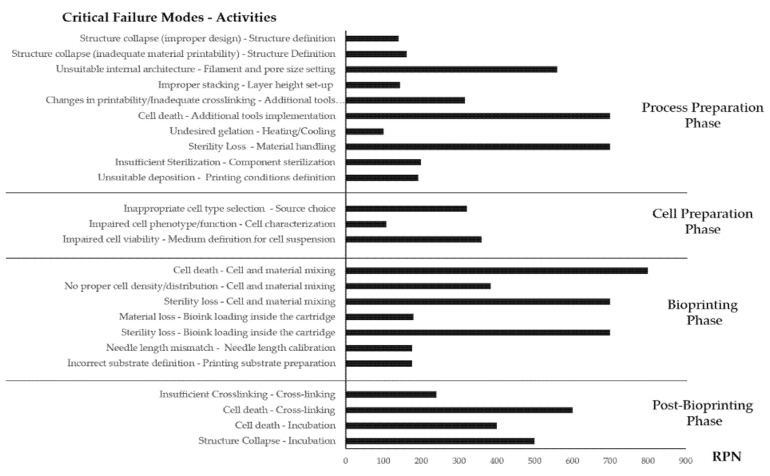
Schematic representation of critical (RPN > 100) failure modes and their related activities together with the established RPN values.

The results highlighted RPN values greater than 100 in many cases. This was expected, as the process was not only new, but also complex, long-lasting, and articulated. The failure modes involving cellular death and/or microbial contamination were identified to have the highest RPN, with nine activities presenting an overall value greater than 400 (Figure 4).

The highest O values were observed for the process aspects related to improper material or cell handling, which represent delicate steps. Especially in the less consolidated mixing process, great care has to be put to avoid cell damage and death, as well as contamination. Other critical activities are the setting of design (such as layer height), printing, and crosslinking conditions, which must be thoroughly optimized to guarantee a proper shape fidelity and stability over the time of the fabricated constructs (as shown in Figure 3 (1)). The highest S values were assigned, aside from the failure modes involving cellular death or contamination, to instrument-related failures (e.g., power outages or system errors), that not only may cause, if prolonged, adverse consequences in terms of cellular death, but may also lead, in the worst cases, to effective damages to the system. This is an often-ignored aspect by most researchers who tend to perform control experiments to identify issues in their process. The FMEA/FMECA also helps to analyze the instrument’s failure possibility and highlight the importance of calibration and regular maintenance as a mitigation action. The highest values of D were related to possible failures involving material deposition and stacking during the printing process, which may be observed in real-time and corrected with minimal time and material waste. Consequences such as cell death or culture contamination, which are difficult to detect before the process ends, will lead to a complete failure of the experiment.

The 3D structure design step is crucial for the fabrication of structures mimicking the properties of native cartilage, in terms of mechanical compliance, pore size and distribution, high level of anisotropy, and zonal organization [13]. Material composition, micro and macro architecture, and 3D rheological and physical properties may strongly affect the process, both in terms of deposition (e.g., pore size and layer height) and the selection and use of appropriate auxiliary tools/crosslinking mechanisms. Natural polymer-based bioinks, such as collagen, due to their low viscosity, tend to present non-optimal shape recovery and self-sustaining capabilities, resulting in some filament spreading phenomena post deposition [22]. This can lead to a reduction of the pore size compared with the theoretical value and an unsuitable stacking performance because of the decreased layer height (as shown in Figure 3 (2A,B)). An optimal design requires considering these aspects and correcting the structure accordingly. In the analyzed process, collagen-based bioinks are thermosensitive gels, and their properties are strongly dependent on pH and temperature. Since a neutral pH is required to perform cell-laden bioprinting processes, a way to improve material printability is to enable temperature control during the process. As demonstrated by previous research in our group, keeping material cartridge at a low temperature improves material flow and avoids clogging, while depositing the material on a heated collector (37 °C) to favor immediate crosslinking, which improves shape retention and stacking capability [36].

Collagen-based biomaterials can also be found in the form of standardized solutions as pre-made bioinks [22], which can be provided in acidic or already neutralized form. While the first case represents a thermally stable and easy to print alternative, it is nonetheless required, before cell addition, to mix the material with an adequate buffer solution to reach a physiological pH. Though, in the analyzed process, the selected collagen formulation was represented by a commercial acidic type I collagen solution, we decided to also mention and take into account, in the biomaterial preparation step, other possible preparation routes that may involve the concentration of collagen solutions or the dissolution of collagen powder into an appropriate medium (usually acetic acid, followed by a neutralization step). To maximize the efficiency of these preparation procedures, additional steps such as stirring or centrifuging may be required to obtain a homogeneous final product.

In the setting of the process, adequate safety procedures must be undertaken to guarantee cell survival. The printing system components (needles, cartridges, and connectors) must be cleaned and sterilized (e.g., autoclaving); the bioprinter must be enclosed in a biosafety cabinet equipped with High-Efficiency Particulate Air (HEPA) filters; all surfaces must be cleaned and disinfected through solvent mixtures as well as UV irradiation. Afterwards, it is required to input the desired printing parameters in the control software. These parameters may be already provided by the producer in the case of commercially available bioinks or may require a preliminary testing phase in the case of lab-made materials. Optimization is therefore performed through the evaluation of the fidelity of the fabricated structures to the designed geometry. Usually, this printing parameter optimization is performed on the biomaterial formulation before cell addition in order to reduce their consumption to a minimum. Slight adaptation may be required during the bioprinting process to match the changes in viscosity of the cell-laden bioinks compared to the pure biomaterial formulation.

Regarding the preparation of the cells to be embedded within the material, RPN values were lower than the other phases, except for a few cases. This happened because the cell culture process is already widely standardized in research laboratories, including ours, and therefore it presents a few issues. The most critical activities with RPNs > 100 were clinical need/cell source evaluation and medium cell suspension definition. The first activities are important for improving the clinical translation of the process: it is important to choose a population of cells able to induce cartilage regeneration when interacting with the biomaterial and upon their placement in the lesion site. Medium definition for cell suspension is another critical activity because balance conditions should be created to favor both cell growth and material stability.

The bioprinting process starts with the preparation of the cell-laden bioink through the mixing of the cells with the biomaterial. This procedure can range from manual techniques (involving gently mixing using a spatula for stronger gels or simple multiple pipetting for less viscous formulations) to more user-friendly systems (dual syringes with ad hoc connectors to mix the materials through back-and-forth injection). Then, mild centrifugation may be required to remove air bubbles trapped within the bioink, which can negatively affect the printing outcome. The whole cell-laden bioink preparation is critical: the acidic form selected for collagen storage implies the need for a neutralization phase with a buffer solution, and an improper neutralization procedure may lead to cell death due to their embedding in a non-suitable environment pH-wise. Moreover, the dual syringe mechanism selected, though presenting advantages in terms of friendliness and mixing performance, may still negatively affect cell survival due to mechanical stress during the mixing phase, which has to be repeated multiple times to guarantee material neutralization and cell distribution homogeneity. Lastly, the embedding process requires cells to be kept in suspension to be mixed with the hydrogel. This addition, combined with the required volume of buffer to neutralize the ink, clearly implies a noticeable variation in the bioink final viscosity and printability, which could adversely affect the dispensing process and final construct fidelity. For this reason, it is highly recommended on the one hand to minimize the cell suspension volume required for mixing, and, on the other, to perform a preliminary testing phase to quickly adapt printing parameters to the modified material formulation.

Once the cell-laden bioink is ready, the preparation of the printing process may start. The formulation is transferred to the printhead, usually through a compatible cartridge that is connected to the system pressure line and the desired needle for extrusion. Then, a calibration of the system is required, generally consisting of the measurement of the selected needle length through automated (via light-based sensors) or manual techniques. Moreover, the printing surface, represented by a glass slide, a well plate, or a Petri dish, is prepared on the instrument collector. This may require a further calibration step to provide the system with the geometrical parameters of the desired printing substrate, which may be through predefined libraries, automated calibration systems, or manual editing. In addition, auxiliary support tools for gelation/crosslinking such as the use of heating/cooling for the substrates/collector need to be enabled with the proper advance to reach stable thermal conditions. Temperature control, for example, represents a key factor in the collagen extrusion process, due to the specific material properties. An excess in cartridge temperature may lead to pre-gelation phenomena that may cause needle clogging and subsequent process failure, or, even if material flow persists, it may require higher pressure values that could be detrimental to cell survival (Figure 3 (4A,B)). On the other hand, lower temperatures of the printing substrate may result in unsuitable deposition and stacking phenomena, due to insufficient or slow thermal gelation processes, resulting in material spreading and loss of stability. After the system initialization, the desired printing instructions are loaded into the instrument control software and the process is performed. Though most bioprinting systems are characterized by a high degree of automatization, operator presence may still be preferable during the first phase of the process in order to quickly identify unexpected issues or unsatisfactory performance (that may be due to improper design or process parameters, errors during calibration procedures, and difference in the formulation batch properties from the expected behavior) and correct them immediately, avoiding the time and material waste, at least until a qualitative inspection of the first layers or scaffolds reveals a suitable outcome.

Post-processing of the sample may be required to improve final construct properties. Different research approaches may exploit different crosslinking mechanisms for collagen-based bioinks. The use of photochemical modifications can be applied to provide increased mechanical performance and stability of the construct and promote chondrogenic differentiation [37]. Another possibility is the use of chemical crosslinking agents [27] which are known to often cause cytotoxicity effects [27] but have been demonstrated to be compatible with cell activity in some cases (use of tannic acid in combination with a cell-laden collagen hydrogel) [38]. In our process, we utilized a thermal gelation method, which exploits the thermosensitive nature of collagen-based bioinks to reach the hydrogel state under physiological conditions (neutral pH and 37 °C) through self-organization of collagen molecules into fibrils. This also allowed performing the incubation step immediately post printing in order to increase cell viability while guaranteeing an improved shape fidelity. Finally, to ensure cell survival, it is critical to perform the whole process in the shortest possible time.

## 5. Conclusions

Our study demonstrates that the FMEA/FMECA is a useful proactive tool to standardize an investigational process in a research lab comprising the 3D extrusion-based bioprinting of an MSC-laden type I collagen bioink process. The perspective of exploiting this simplified structure could allow to recapitulate more complex bioprinting processes and strategies, including the use of different bioinks or heterogenous cell cultures to more accurately mimic native tissue properties. This proactive analysis could open interesting perspectives also for the evaluation of the product quality assessment process as part of the analysis itself, whereby many failure models can occur. Employing this proactive risk analysis in a preliminary stage can avoid material and time waste for scientists by providing a technical guideline. It is important to highlight early-stage issues and critical steps alongside possible mitigation measures to minimize failure chances. Controlling the causes of variability will enable to improve the repeatability of the results and to implement standardized procedures to be more easily translated into clinical use to target cartilage regeneration. The possibility of bridging the gap between research and clinic through this tool will allow faster progress of knowledge and investigations as well as a saving of time and money.

## Figures and Tables

**Table 1:** Occurrence scale. 1–10 ranking correlates with the progressive increase in the likelihood of occurrence of a failure mode during bioprinting.

Value	Definition	Interpretation
1–4	Impossible/infrequent	It is virtually impossible or rare to occur
5–6	Occasional/moderate	It occurs a few times
7–8	Frequent	It often occurs
9–10	Very frequent/certain	It occurs several times or will certainly occur within a short time

**Table 2:** Severity scale. The four groups correlate with the increased bad potential effect of the considered failure modes.

Value	Definition	Interpretation
1–4	Low	Technical problems during activity, without implications on product quality
5–6	Moderate	Minor problems slightly lowering the product quality
7–8	High	Major problems affecting product quality
9–10	Dangerous	No product

**Table 3:** Detection scale. 1–10 ranking correlates with the progressive decrease in the likelihood that the failure will be detected before the bioprinting process ends.

Value	Definition	Interpretation
1–4	Certain/ very high	Errors are usually intercepted before affecting process
5–6	Medium/high	Errors are trapped unless operator inattention or specific measures are in place to detect potential problems
7–8	Low	Issues are occasionally detected during the activities
9–10	Almost zero	Only an external action or intervention other than the routine practice may highlight the error or there is no chance to detect the problem

**Table 4:** FMEA/FMECA of the pre-bioprinting phase and process preparation sub-phase.

Step	Activity	Failure Mode	Potential Cause	Possible Consequences	O	S	D	RPN	Mitigation Actions
3D structuredesign	Structure definition	Collapse	Improper design for fiber deposition (complex structures, overhanging segments)	Failure to replicate the desired shape Structure loss Unsuitable bioprinting results	4	7	5	140	Correct orientation of the shape Generate software 3D preview of the designed object
	Structure definition	Collapse	Inadequate material Printability	Failure to replicate the desired shape Structure loss Unsuitable bioprinting results	6	9	3	162	Use of support materials Adapt material formulation to improve rheological properties Perform acellular material deposition tests to adapt process parameters
	Filament and pore size setting	Unsuitable internal architecture	A mismatch between the designed filament and fabricated one (use of the different needle, unsuitable material flow) Improper structure definition (pore size, fiber diameter, deposition pattern)	Unsuitable nutrient supply/cell death (closed porosity) Inadequate mechanical properties/structure integrity loss (excess porosity)	6	8	2	96	Generate software 3D preview of the designed object. Double-check design parameters about selected consumables before process launch
3D structure design	Filament and pore size setting	Unsuitable internal architecture	Improper structure definition (pore size, fiber diameter, deposition pattern)	Failure to mimic properties of native tissues to induce cell differentiation and ECM production	7	8	10	560	Pilot studies to test different 3D architectures for selecting the best design for cell growth and cartilage tissue production
	Layer height set-up	Improper stacking	Layer fusion (low layer height) Missing deposition (excessive layer height)	Structure loss Material accumulation on needle tip without structure formation	9	8	2	144	Optimization of printing process parameters using acellular material to establish adequate layer height for optimal stacking
	Additional tools (heat, UV) implementation	Changes in printability Inadequate crosslinking	Cartridge and/or needle tip temperatures unsuitable for ink flow Platform temperature unsuitable for material crosslinking/gelation Improper dosage of UV irradiation	Improper gelation (Needle clogging, excess deposition) structure collapse/shape loss	7	9	5	315	Double-check additional tools settings before process launch Perform preliminary dispensing and crosslinking tests on acellular material Wait additional time, once temperature setpoints are reached, to guarantee thermal equilibrium conditions
3D structure design	Additional tools (heat, UV) implementation	Cell death	Cartridge temperature inadequate for cell survival Improper dosage of UV irradiation	No biological response Experimental failure	7	10	10	700	Evaluate through scientific literature threshold values of cell exposure to crosslinking agent Wait for additional time once temperature setpoints are reached, to guarantee thermal equilibrium conditions
Biomaterial preparation	Dissolving in liquid medium	Incomplete dissolution	Use of ineffective solvent Solution Saturation Improper dissolution procedure	Sedimentation Decreased bioactive concentration Unsuitable rheological/printability properties	4	6	2	48	Obtain solubility data (max concentration/solvent compatibility) from scientific literature or material supplier Follow operative instructions for material dissolution provided by the supplier
	Stirring	Inhomogeneous distribution	Insufficient time Incorrect procedure (temperature, material addition ratio, speed)	Formation of aggregates/lumps Decreased bioactive concentration Unsuitable rheological/printability properties	4	6	2	48	Optimize material dissolution parameters preliminarily with small material amounts Follow the defined operative instructions
Biomaterial preparation	Mixing	Unsuitable rheology	Incorrect mixing ratio	Unsuitable rheological/printability properties	3	6	5	90	Request optimized mixing ratio to the supplier or identify a suitable starting ratio from scientific literature Perform preliminary tests on material dispensing and deposition to evaluate formulation printability
	Heating/cooling	Undesired gelation	Material kept above/below required temperature for a prolonged time during the preparation phase	Manipulation issues Needle clogging	5	5	4	100	Follow operative instructions provided by the manufacturer Check reaching and keeping of the temperature setpoints throughout the process
	Centrifuging/ vortexing	Material inhomogeneity	Incorrect settings (time, speed) Unforeseen material Properties	Unsuitable deposition/needle clogging (air bubbles presence) Phase separation (sedimentation due to excess centrifuging) Undesired gelation	4	6	2	48	Check for available optimized parameters set from scientific literature or distributors Visually inspect centrifuged–vortexed solutions for signs of sedimentation/gelation
Biomaterial preparation	Material handling	Sterility loss	Improper handling procedure Non-sterile environment	Culture contamination Experiment failure Material waste	7	10	10	700	Follow sterile work procedures Visual inspection to identify sterility losses of required consumables (e.g., autoclave bags integrity) Regular maintenance/calibration of biological safety hoods
Process set-up	Disposable selection and preparation	Disposable unavailability	Out of stock Mismatch between selected disposables and design Disposable loss due to issues during the process	Incorrect process parameters (needle mismatch) Process interruption (lack of required disposables)	3	5	5	90	Keep an updated stock register Proactively reorder disposables close to consumption/expiry Plan 2 backup disposable sets for each experiment
	Sterilization of the required components—printing environment	Insufficient sterilization	Unsuitable sterilization method Sterilization Procedure failure	Culture contamination Experiment failure Material waste	2	10	10	200	Identify compatible sterilization procedures Follow operative instructions for sterilization procedures. Visual inspection to identify unforeseen failures (e.g., autoclave bags integrity)
Process set-up	Printing conditions definition	Unsuitable deposition	Improper process parameters	Shape loss Variation in the internal architecture Structure collapse Process interruption (needle clogging)	6	8	4	192	Acellular optimization of printing process First tests with low amounts of cell-laden material Check that desired process conditions have been reached and stable before running the process

**Table 5:** FMEA/FMECA of the pre-bioprinting phase and cell preparation sub-phase.

Step	Activity	Failure Mode	Potential Cause	Possible Consequences	O	S	D	RPN	Mitigation Actions
MSC selection type	Clinical need evaluation/Source choice	Inappropriate cell type selection	Inadequate cell response	Impaired chondroprotective cell potential	5	8	8	320	Definition of new selection criteria
MSC culture	Cell isolation	Microbial contamination Cell death/low cell growth Non-idoneous cell number/passage Impaired cell phenotype	Human error, low instrument performance Improper culture conditions Sample variability	No adequate cell availability to continue the process Delay in experimental plan	4	10	2	80	Periodic instrument controls (calibration) Optimization of culture conditions Definition of strict criteria for sample processing Personnel training
	Cell characterization	Microbial contamination Cell death/low cell growth Non-idoneous cell number/passage Impaired cell phenotype	Human error, low instrument performance Improper culture conditions Sample variability	No adequate cell availability to continue the process Delay in experimental plan	5	9	2	90	Periodic instrument controls (calibration) Optimization of culture conditions Definition of stricter criteria for sample processing Personnel training
MSC culture	Cell expansion	Microbial contamination Cell death/low cell growth Non-idoneous cell number/passage Impaired cell phenotype	Human error, low instrument performance Improper culture conditions Sample variability	No adequate cell availability to continue the process Delay in experimental plan	8	6	2	96	Periodic instrument controls (calibration) Optimization of culture conditions Definition of stricter criteria for sample processing Personnel training
MSC suspension preparation	Cell harvest	Low cell growth	Human error Improper culture conditions Sample variability	Lower than planned yield A high amount of cell death Delay in the experimental plan	4	6	2	48	Optimization of culture conditions Definition of more stringent criteria for sample processing Personnel training
	Cell characterization	Impaired cell phenotype/function	Improper culture conditions	Wrong/non-idoneous cell population	6	9	2	108	Periodic instrument controls (calibration) Optimization of culture conditions Optimization of culture conditions
	Medium definition for cell suspension	Impaired cell viability	Improper culture conditions	High cell death during post-printing phase	6	10	6	360	Optimization of culture medium for cell suspension

**Table 6:** FMEA/FMECA of Bioprinting phase.

Step	Activity	Failure Mode	Potential Cause	Possible Consequences	O	S	D	RPN	Mitigation Actions
Bioink preparation	Cell and material mixing	Cell death	Excess mechanical stress Prolonged exposure to adverse conditions Improper handling Altered physical properties of the bioink (low pH)	No biological response Experiment failure Material waste Cytotoxicity	8	10	10	800	Follow mixing procedures provided by the manufacturer or optimized in the lab Adopt ad hoc cartridge mixing systems
	Cell and material mixing	No proper cell density/ distribution	Improper cell signaling within the biomaterial	No adequate cross-talk between cells and scaffold	8	8	6	384	Pilot studies for evaluating different cell densities by defining the best biological responses
	Cell and material mixing	Sterility loss	Improper handling	Culture contamination Experiment failure Material waste	7	10	10	700	Follow mixing procedures provided by the manufacturer or optimized in the lab Follow operative instructions concerning sterilization procedures
Bioink preparation	Bioink loading inside the cartridge	Material loss	Improper handling	Material waste Process interruption	6	10	3	180	Follow operative instructions concerning bioink handling
	Bioink loading inside the cartridge	Sterility loss	Improper handling	Culture contamination Experiment failure Material waste	7	10	10	700	Follow operative instructions concerning sterilization procedures
Printing preparation	Needle and pressure line connection	Material spill	Improper connection	Insufficient flow Material waste Process interruption	2	5	3	30	Follow operative instructions for bioink handling Perform preliminary extrusion tests in manual dispensing mode with low-pressure values
	Needle length calibration	Needle length mismatch	Incorrect calibration procedure Different printing substrate	Improper deposition and stacking Needle crash Substrate damage	5	7	5	175	Follow calibration procedures provided by the manufacturer Adopt, where possible, automated calibration systems Manually verify calibration by prompting needle movement to the start position or simulate printing without pressure
Printing preparation	Printing substrate preparation	Incorrect substrate definition	Substrate change between processes Operator error Incorrect calibration procedure	Improper deposition and stacking Needle crash Substrate damage Process interruption	5	7	5	175	Follow calibration procedures provided by the manufacturer Check the selected substrate in the printer software Manually verify calibration by prompting needle movement to the start position or simulate printing without pressure
Bioprinting process	Design file loading in the HMI	Wrong design loading	File change or update between processes Outdated file version Operator error	Unsuitable deposition Structure loss Needle crash Process interruption	2	7	2	28	Implement efficient, compartmentalized files storage procedures Do not store multiple versions of the files locally, use online backup or external supports Visually check the loaded filename before every process start
	Printing process execution	Process interruption	System error	Time loss Material waste Needle crash Substrate damage	1	10	1	10	Constant software update and hardware maintenance
Bioprinting process	Printing process execution	Process interruption	Power interruption	Time loss Material waste Needle Crash Substrate Damage	1	10	1	10	Connection of the bioprinter to electrical backup systems (UPS)
	Printing process execution	Process interruption	Improper procedure	Time loss Material waste Needle crash Substrate damage	3	10	1	30	Follow operative instructions provided by the manufacturer

**Table 7:** FMEA/FMECA of the post-bioprinting phase.

Step	Activity	Failure Mode	Potential Cause	Possible Consequences	O	S	D	RPN	Mitigation Actions
Post bioprinting	Cross-linking	Insufficient crosslinking	Insufficient concentration of initiator/chemical crosslinker Insufficient exposure to crosslinking mechanisms (light irradiation times) Improper crosslinking conditions (temperature, light wavelength)	Shape loss Structure collapse	6	8	5	240	Optimized crosslinking procedures based on technical data sheet and literature research Preliminary testing with non-cellularized materials to evaluate crosslinking efficiency
		Cell death	Prolonged cell exposure to adverse conditions (UV, chemical, temperature)	No biological response Experiment failure Material waste	6	10	10	600	Minimize process time Evaluate through scientific literature threshold values of cell exposure to crosslinking agent
	Incubation	Cell death	Prolonged exposure to cell survival adverse conditions Inadequate nutrient supply for cell growth	No biological response Experiment failure Material waste	4	10	10	400	Minimize fabrication window (printing + crosslinking time) Implement an intermediate incubation step before performing post-printing evaluations
		Structure collapse	Lack of stability under in vitro culture conditions	Material waste Experiment failure	5	10	10	500	Preliminary testing with non-cellularized materials to evaluate crosslinking efficiency and stability under in vitro culture conditions

## Data Availability

Not applicable.
